# Variation of Pneumococcal Pilus-1 Expression Results in Vaccine Escape during Experimental Otitis Media [EOM]

**DOI:** 10.1371/journal.pone.0083798

**Published:** 2014-01-08

**Authors:** Marisol Figueira, Monica Moschioni, Gabriella De Angelis, Michèle Barocchi, Vishakha Sabharwal, Vega Masignani, Stephen I. Pelton

**Affiliations:** 1 Section of Pediatric Infectious Diseases, Maxwell Finland Laboratory for Infectious Diseases, Boston Medical Center, Boston, Massachusetts, United States of America; 2 Novartis Vaccines and Diagnostics, Siena, Italy; Instituto Butantan, Brazil

## Abstract

The pneumococcal Pilus-1 enhances attachment to epithelial cells in the respiratory tract and subsequent invasion. Pilus-1 expression is bi-stable and positively regulated by the RlrA transcriptional regulator. To delineate the role of pilus-1 in Experimental Otitis Media (EOM), we evaluated colonization and disease due to a *Streptococcus pneumoniae* (SP) wild type strain (Taiwan19F-14 wt) and its otherwise isogenic pilus-1 and pilus-2 deficient mutant (Taiwan19F-14 ΔPI-1/PI-2-) as well as potential for a chimeric protein (RrgB321) vaccine candidate for prevention of middle ear (ME) disease.

**Methods:**

Chinchillas were challenged intranasally with either Taiwan19F-14 wt or Taiwan19F-14PI-1/PI-2 deficient mutant. ME status was assessed and direct cultures performed. New cohorts of animals were immunized with RrgB321 or alum. Intranasal challenge with Taiwan19F-14 wt [erythromycin susceptible E(S)] was performed. Subsequently, a second cohort of animals was immunized and challenged with either Taiwan19F-14 wt or a Pilus-1 over-expressing mutant [Taiwan19F-14+pMU1328_Pc-rlrA mutant; E resistant (R)] strain. Pilus-1 expression was analyzed in SP isolated from nasopharynx (NP) and ME fluids by flow cytometry.

**Results:**

Culture positive EOM developed following challenge with either wild type SP (Taiwan19F-14) or its pilus-1 deficient mutant. Culture positive EOM developed following challenge with wild type in both RrgB321 immunized and control animals. Pilus-1 expression in ME fluids was significantly higher in controls compared to immunized chinchillas. In second cohort of immunized and control animals challenged with the over-expressing Pilus-1 mutant, delayed development of EOM in the immunized animals was observed. Pneumococci recovered from ME fluid of immunized animals were no longer E(R) signifying the loss of the pMU1328_Pc-rlrA plasmid.

**Conclusion:**

Pneumococcal pilus-1 was not essential for EOM. Regulation of Pilus-1 expression in ME fluids in the presence of anti RrgB321 antibody was essential for survival of *S. pneumoniae*. Pneumococci have evolved mechanisms of regulation of non-essential surface proteins to evade host defenses.

## Introduction

Several virulence factors are known to be responsible for *S. pneumoniae* pathogenesis and ability to infect the human host. One of these factors is the pilus-1, a long, multimeric filament composed of covalently linked subunits. Thirty percent of pneumococci have the genetic capacity to produce pili as reflected by the presence of the pilus islet 1 [PI-1], of which three different genetic clades have been identified [1](. The pneumococcal pilus-1 consists of the RrgB protein which forms the backbone and two ancillary proteins, RrgA and RrgC. RlrA regulated pilus-1 expression is biphasic; in fact, in PI-1 positive strains, there are always pilus expressing and pilus not expressing bacteria within the bacterial population, whose proportion is not dependent on the genetic characteristics of the different strains and has the capacity to vary with *in vivo* infection [2] [1] [3]. However, environmental factors that facilitate and modulate expression are currently poorly understood.

The role of pilus-1 is traditionally thought to be related to adherence as well as virulence, yet *in vitro* there is no significant difference in pneumococcal binding to matrix proteins between pilus-1 deletion mutants and wild type strains. Furthermore, only non-statistically significant, minor differences are observed in binding assays to activated lung epithelial cells between wild type and pilus-1 deficient mutants, and only a small, statistically significant difference in nasal colonization density is observed following intranasal challenge of BALB/c mice. Rosch and colleagues reported divergent outcomes between wild type and pilus negative mutants in an intratracheal challenge model with markedly lower lung bacterial loads in mice challenged with the pilus-1 negative mutants. They hypothesized a lung specific role for pili in the pathogenesis of pulmonary disease. Their observations are consistent with the protection against pulmonary disease following immunization of mice with a pilus-1 protein vaccine antigen, the RrgB321 chimera, consisting of the three existing RrgB variants in a head to tail organization [4].

Interestingly, alongside PI-1, PI-2 (pilus islet 2) has been identified in a smaller proportion of pneumococcal strains (about 16%), a minority of which are positive for the presence of both islets. However, unlike pilus-1, pilus-2 is evenly expressed in PI-2 positive strains and its expression is thought to have a marginal contribution in bacterial adhesion to epithelial cells [5].

To delineate the role of pilus-1 in otitis media we used the chinchilla of experimental otitis media model (EOM) and evaluated colonization and disease due to the wild type *Streptococcus pneumoniae* strain Taiwan19F-14 wt and its otherwise isogenic Taiwan19F-14 ΔPI-1/PI-2 deficient mutant as well as the potential for RrgB321 to prevent middle ear (ME) disease.

## Materials and Methods

### *Streptococcus pneumoniae* strains and culture conditions

The names and the genetic characteristics of the *Streptococcus pneumoniae* (SP) strains used in this study are reported in Table 1. Bacteria were grown at 37°C under 5% CO_2_ atmosphere on Tryptic Soy Agar (TSA, Difco) plates containing colistine 10 mg/l, oxolinic acid 5 mg/l, and 5% defibrinated sheep blood; erythromycin (100 µg/mL) and/or kanamycin (500 µg/mL) were added to the medium when necessary. Following overnight (o.n.) growth on agar plates, bacteria were used to inoculate liquid cultures in THYE (Difco). Growth was carried out statically at 37°C under 5% CO_2_ atmosphere until A_600_ = 0.25 was reached. Bacteria were then harvested by centrifugation at 3,500 r.c.f. for 20 min at 4°C, resuspended in saline and used within 1 h for intranasal administration. The colony forming units (CFUs) per ml were determined by seeding serial dilutions on plates and counting the colonies after overnight growth [6].

**Table 1:** Strains used in this study.

Stain name	Source	Country	Serotype	ST (CC)	PI-1	PI-2	Ery^R^	Kan^R^	Reference
Taiwan19F-14	PMEN clone	Taiwan	19F	236 (271)	yes	yes	2	no	[17]
Taiwan19F-14 ΔPI-1/PI-2	genetically modified		19F	236 (271)	no	no	100	yes	[5].
Taiwan19F-14 pMU1328_Pc_rlrA	genetically modified		19F	237 (271)	no	no	100	no	[3]

### Generation of Taiwan19F-14 derivatives

Taiwan19F-14ΔPI-1/PI-2 isogenic mutant was generated by allelic exchange as reported previously [5]. Briefly, fragments of approximately 500 bp upstream and downstream the target genes were amplified by PCR and spliced to a kanamycin or erythromycin antibiotic cassette by using overlap extension PCR; the obtained PCR fragment was cloned into pGEMt (Promega) and the obtained plasmid transformed in Taiwan19F-14 wt strain with conventional methods. Bacteria were plated on selective blood-agar plates (kanamycin 500 µg/ml; erythromycin 1 µg/ml). The presence of the isogenic mutations was confirmed by PCR and the absence of pilus-1 and pilus-2 expression confirmed by western blot analysis.

**Figure 1:**
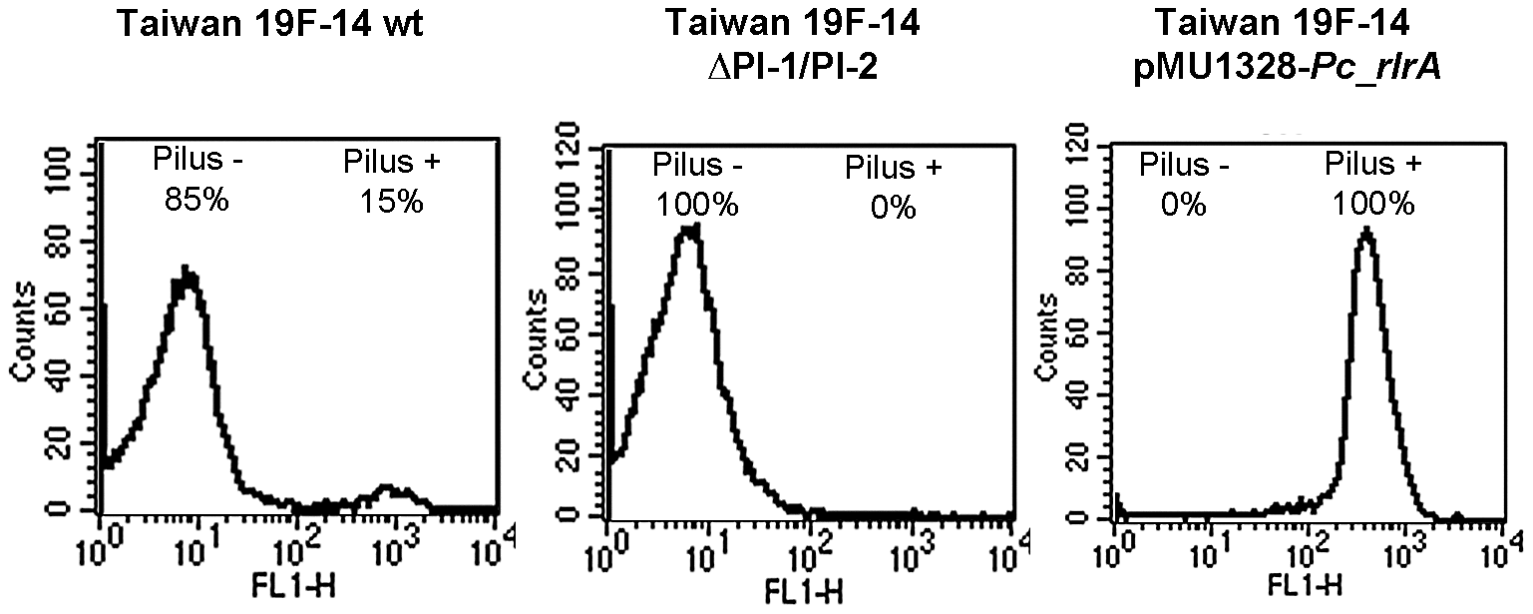
Evaluation of pilus expression on surface of *Streptococcus pneumoniae*. Flow cytometry on SP Taiwan19F-14 wt and its derivatives (ΔPI-1/PI-2 mutant and pMU1328_*Pc-rlrA* + strain) performed with pAb against RrgB. The percentage of pilus positive and negative bacteria for each strain was reported.

The Taiwan19F-14pMU1328-*Pc_rlrA* used in this work was obtained as previously described [3]. Briefly, the *rlrA* gene was amplified on the TIGR4 genomic DNA while the erythromycin resistance (ermB) constitutive promoter (*Pc*) was amplified on the pMU1328 plasmid containing an erythromycin resistance marker. The obtained PCR products were digested with the appropriated restriction enzymes and cloned into the complementation plasmid pMU1328. The Taiwan19F-14 strain was then transformed with conventional methods [7] and selection of transformed bacteria performed on TSA plates supplemented with 100 µg/ml erythromycin [8]. Flow Cytometry was used to demonstrate that 100% of the Taiwan19F-14 pMU1328-*Pc_rlrA* displayed pilus on their surface (Fig. 1). The evaluation of possible plasmid loss was made by comparing CFUs growing on plates supplemented either with 100 µg/ml erythromycin (selective) or 1 µg/ml erythromycin (non-selective).

### Animals

An experimental chinchilla model of acute experimental otitis media (EOM) was used [9]. Adult chinchillas (*Chinchilla lanigera*) were obtained from Ryerson's Chinchilla Ranch (Plymouth, OH).

### Ethics Statement

The Institutional Animal Care and Use Committee at Boston University Medical Center approved our animal care protocols as being consistent with humane treatment of laboratory animals and with standards set forth in the Guide for the Care and Use of Laboratory Animals and the Animal Welfare Act (Animal Welfare Assurance approval number A-3316-01). Thus, our study was carried out in strict accordance with the recommendations in the Guide for the Care and Use of Laboratory Animals of the National Institutes of Health.

### Immunization Protocol

Animals were separated into two groups, one for immunization with RrgB321 adjuvanted with alum and the other with adjuvant alum alone. The immunized group received 50 µg of RrgB321 along with alum subcutaneously and the control group alum on days 1, 15 and 45. Pre-immunization sera were obtained prior to first immunization and post-immunization sera were obtained 10 days after third vaccine dose.

### RrgB321 recombinant protein expression and purification

RrgB321 recombinant protein was produced as previously reported [10]. Briefly, standard recombinant DNA techniques were used to construct plasmids expressing the RrgB321 chimera, consisting of the three full length RrgB variants in a head to tail organization and separated by a six amino acid linker (Gly-Ser-Gly-Gly-Gly-Gly). *RrgB* open reading frames were amplified by PCR from chromosomal DNAs of *S. pneumoniae* TIGR4 (*rrgB* clade I), 6B SPEC (*rrgB* clade II) and 35 SME 15 (*rrgB* clade III) by using specific primers. The obtained PCR fragments were digested with the appropriated restriction enzymes and ligated into the C-terminal 6×His-tag expression vector pET21b+ (Novagen). The resulting plasmids were confirmed by DNA sequencing and then transformed into competent *E. coli* BL21 DE3 star (Life technologies). The protein was purified by metal chelate affinity chromatography on His-Trap HP columns (GE Healthcare). Pooled fractions containing the purified protein were dialyzed on phosphate-buffered saline (PBS) and stored at −80°C until further use.

### Experimental Otitis Media

To delineate the role of pilus-1 in EOM, chinchillas were challenged intranasally with either Taiwan19F-14 wt or a Taiwan19F-14 ΔPI-1/PI-2 deficient mutant. Twenty four hours after inoculation nasopharyngeal (NP) washes were performed to confirm colonization. On day 4 after inoculation, NP washes (NW) were done and barotrauma was used to create negative pressure as previously described [11]. Daily tympanometry and otomicroscopy were performed to monitor the presence of middle ear infection identified by erythema and bulging tympanic membrane. Once an abnormality was identified, the middle ear cavity was accessed as described previously [12]. Direct culture of middle ear was obtained with a calcium alginate swab and immediately streaked onto a blood agar plate. Middle ear fluid (MEF) when present was obtained and if MEF was absent, the middle ear was flushed with HBSS and then aspirated to determine density of infection [6], [13]. Middle ear culture was obtained at designated time points until the middle ear cultures were sterile on two consecutive samples. Animals immunized with RrgB321 (50 µg) or alum were challenged intranasally with Taiwan19F-14 or Taiwan19F-14+pMU1328_*Pc-rlrA* mutant followed by barotrauma. Nasal washes and MEF culture for *S. pneumonia*e were performed as described above. Pilus-1 expression was analyzed in SP isolated from nasopharynx (NP) and ME fluids by flow cytometry (*see* below).

### Enzyme-Linked Immunosorbent Assay (ELISA)

Serotype specific responses were measured against antigen RrgB321 by modified Enzyme-Linked Immunosorbent Assay. Briefly, flat bottom micro titer plates, Nunc 96 well PolySorp, item #475094 were coated with a 2 µg/ml of RrgB321 in phosphate-buffered saline pH 7.4 (PBS) and left overnight at 4°C. Plates were washed at each step with 1% BSA-1×PBS 0.1% Tween 20, blocked with 2% BSA-1×PBS 0,1% Tween 20. Pre and post-immune chinchilla sera were serial diluted with 1× PBS/0.1% Tween 20; 100 µl were added to wells and incubated at 20°C (room temperature) for 1 h. Once washed three times, wells containing chinchilla samples were incubated with rabbit anti chinchilla IgG secondary antibody at 1∶2000 dilutions and incubated as above. Wells containing chinchilla sera were incubated with Horse Radish Peroxidase (HRP) conjugated goat anti-rat IgG at 1∶1000 dilutions (Southern Biotech.) for 1 hour. Plates were washed, then filled with 100 µl of substrate solution *2-2-Azino-bis (3-ethylbenzothiazoline-6-sulfonic acid)*, incubated and read at 30 and 60 min at 405–690 nm. The results were reported as endpoint geometric mean titers defined as three times the baseline sera optical density.

### Flow cytometry (FC)

Bacteria used as input for intranasal inoculation or recovered form MEF and nasal washes of the chinchillas at different time points post-challenge were plated directly from a frozen glycerol stock. Following growth on TSA/blood plates, bacteria were recovered and stained with rabbit primary antibodies (final dilution 1∶300) [3], and then with a FITC-conjugated secondary anti rabbit antibody (final dilution 1∶100) (Jackson Laboratories). Bacteria were then fixed with 2% paraformaldehyde and bacterial staining was analyzed with a FACS-Calibur cytometer (Becton Dickinson). For each sample 10,000 events were recorded and the percentage of pilus-positive bacteria within each sample was estimated with the CellQuest software (Becton Dickinson).

### Statistical Methods

The mean bacterial density were compared across two groups, RrgB321 immunized and unimmunized animals. The geometric mean bacterial density was calculated for each organism from nasopharyngeal samples on day 1, 4 and 6 after inoculation and on MEF samples on day 6, 8–10 and 13 after inoculation. Differences in the mean were compared using t-tests. In all analyses, p values of <0.05 were considered to be statistically significant.

## Results

### 1. Nasopharyngeal colonization and middle ear challenge with wild type and PI-1/PI-2 mutant

**Figure 2:**
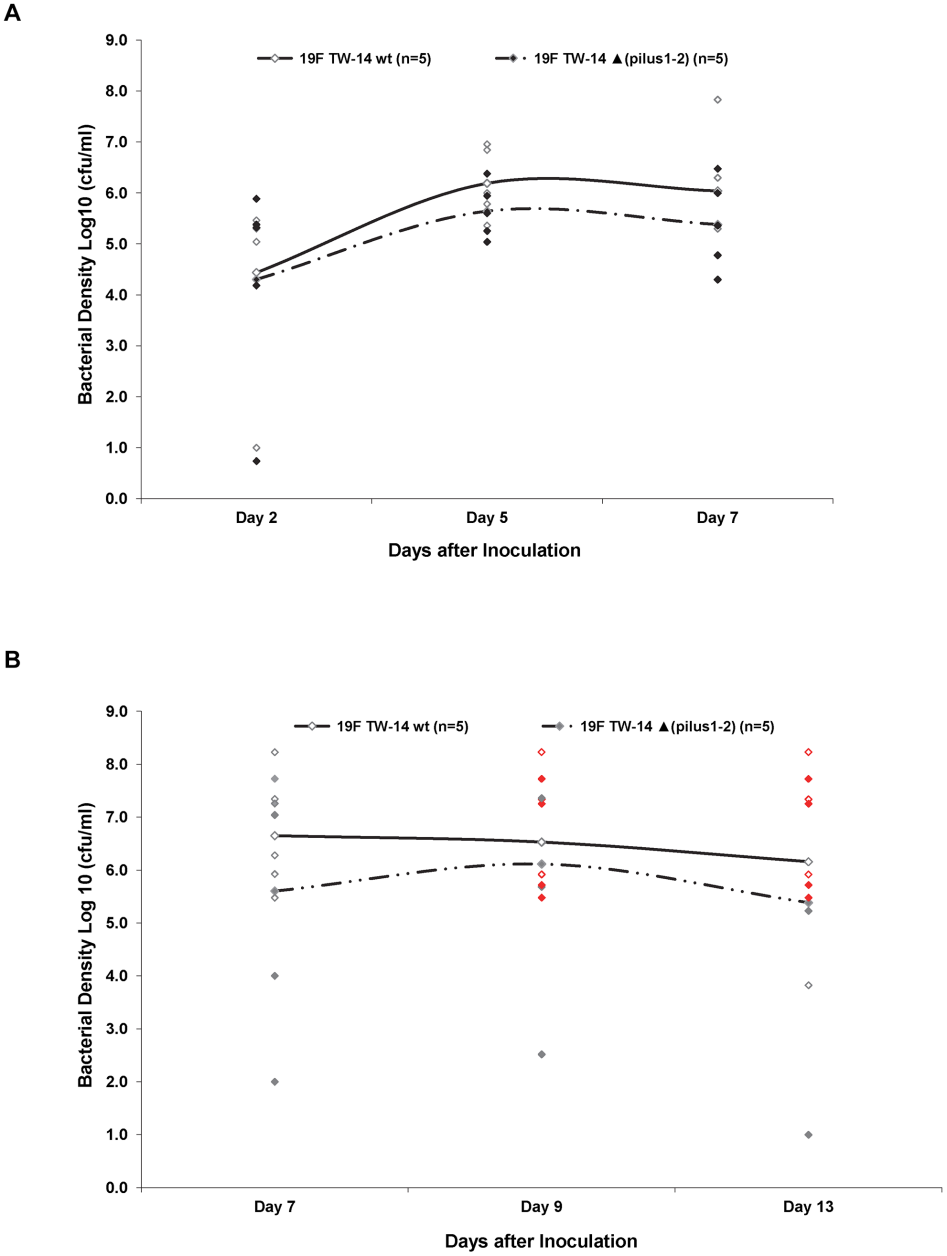
Nasopharyngeal colonization and middle ear challenge with SP Taiwan19F-14 wt and PI-1/PI-2 mutant. Density of colonization in nasopharynx (A) and middle ear fluid (B) in chinchillas challenged with SP Taiwan19F-14 wt (pilus +) or with Taiwan19F-14 ΔPI-1/PI-2 mutant.

Following NP inoculation of ∼5E6 CFU/100 µl of Taiwan19F-14wt (pilus +) and its otherwise isogenic Taiwan19F-14 ΔPI-1/PI-2 mutant, all animals were found to be colonized with the respective inoculated strain and no difference in the bacterial density was observed between the two strains [p>0.05]. (Fig. 2A) Culture positive ME disease with high bacterial density developed in 100% of animals challenged with wild type (pilus +) and the isogenic Taiwan19F-14 ΔPI-1/PI-2 SP following barotrauma (Fig. 2B) No difference in the proportion of animals developing culture positive middle ear disease or middle ear bacterial density between groups was observed.

**Figure 3:**
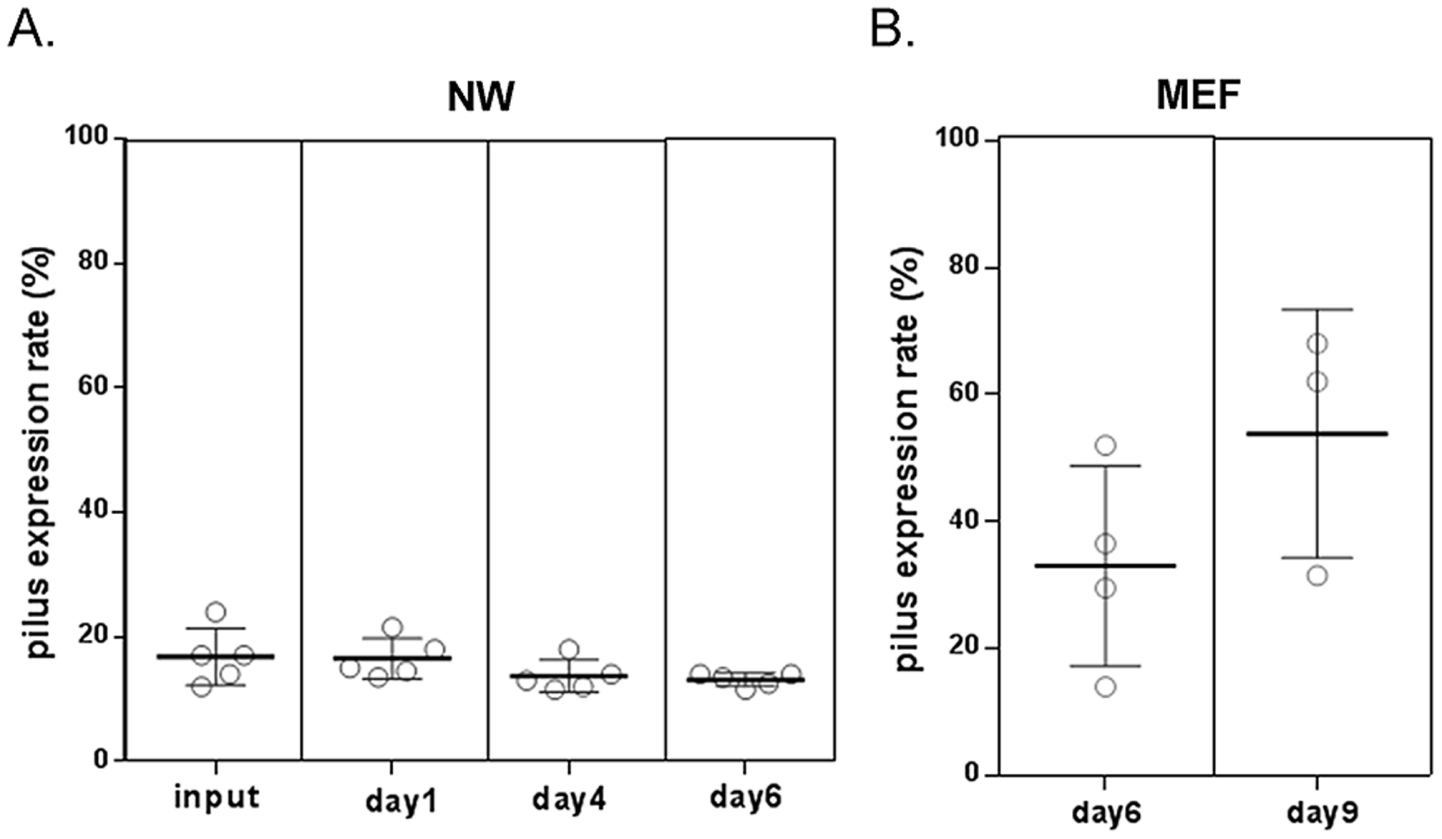
Pilus expression of SP Taiwan19F-14 wt in nasal and middle ear washes compared to inoculum. Flow cytometry analysis of Pilus-1 expression in nasal wash (NW) [A] and middle ear Fluid (MEF) [B] following challenge with SP Taiwan19F-14 wt.

Pilus-1 expression for wild type 19F recovered from the nasopharynx and middle ear was compared with the inoculum to better understand the regulation of pilus-1 expression at each of these sites. Pilus-1 expression in the nasopharynx was comparable between nasal washes and the inoculum (Fig. 3A), while it was increased in the middle ear compared to both the nasopharynx and inoculum (fig. 3A and 3B).

### 2. Immunization with RrgB321 chimera

#### a. Immune response to RrgB321

Animals immunized with RrgB321 demonstrated increased IgG titers compared to controls. The geometric mean endpoint titers were 9700 in the RrgB321 recipients; no antibody to RrgB321 was detected in pre-immune or post immune sera in controls.

#### b. Challenge in RrgB321 immunized animals and controls with wild type SP

**Figure 4:**
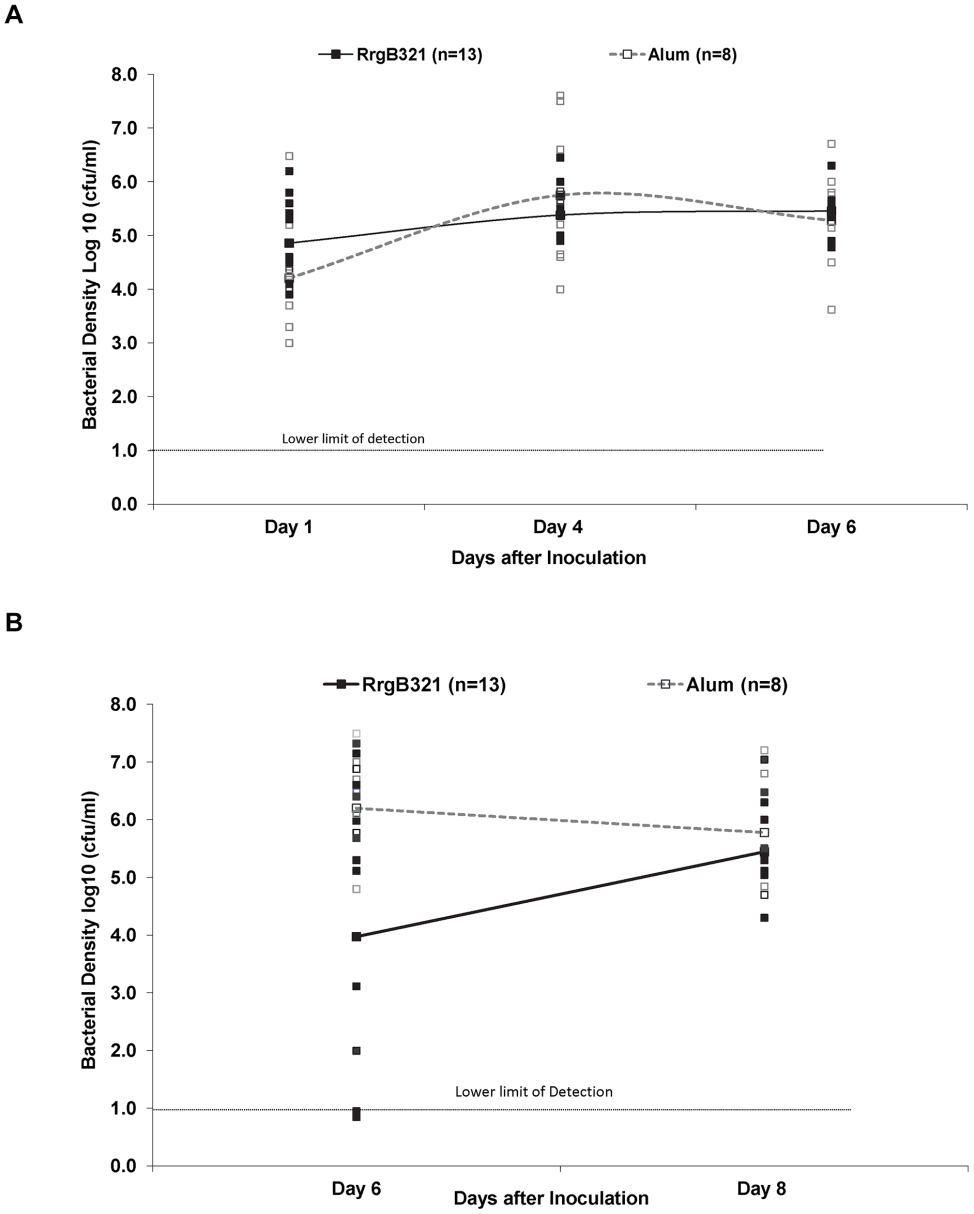
Outcome of challenge with SP Taiwan19F-14 wt in RrgB321 immunized and control animals. Density of colonization in nasopharynx (a) and middle ear fluid (b) in RrgB321 immunized and control chinchillas challenged with SP Taiwan19F-14 wt (pilus +).

Following NP challenge with erythromycin susceptible (S) wild type, we observed colonization in both RrgB321 immunized and control animals (Fig. 4A.) No reduction in bacterial density was observed in immunized animals. Culture positive ME disease was observed in 10 (77%) RrgB321 immunized and all 8 (100%) control animals following NP challenge and subsequent barotraumas (Fig. 4B). Transiently the density of ME infection appeared lower in the immunized animals [1.9E5 versus 1.9E3; p<0.03]; however, by day four following barotraumas (day 8 overall) no differences in density was observed.

#### c. Pilus expression in wild type Taiwan19F-14 in RrgB321 immunized animals

**Figure 5:**
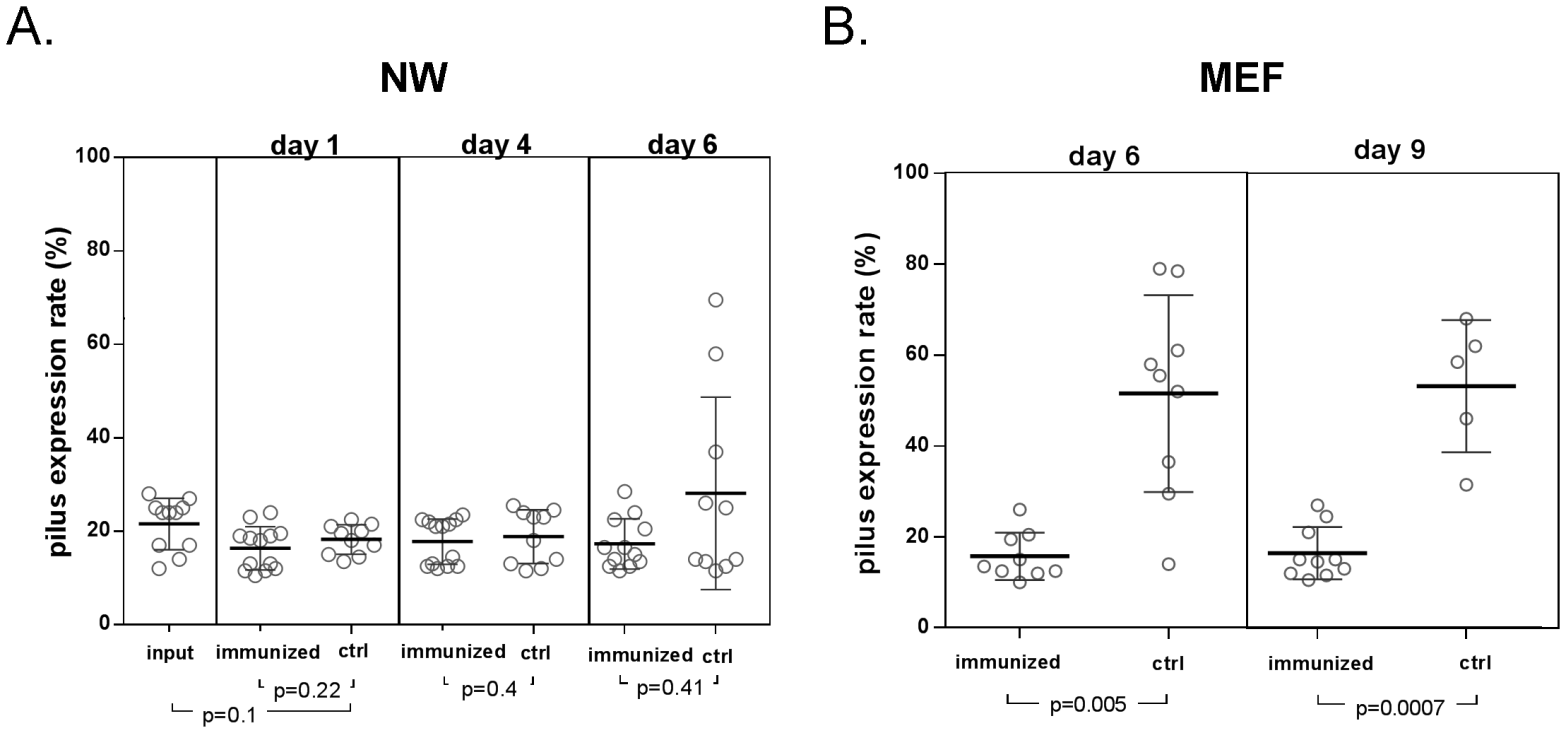
Pilus expression of SP Taiwan19F-14 wt in nasal and middle ear washes of RrgB321 immunized and control animals. Flow cytometry analysis of Pilus-1 expression in nasal wash (NW) [A] and middle ear Fluid (MEF) [B] in RrgB321 immunized and control animals following challenge with SP Taiwan19F-14 wt.

As shown in figure 5A and 5B, flow cytometry studies demonstrated that pilus expression in wild type 19F Tw 14 in the nasopharynx was comparable to that of the inoculum as grown to mid log phase in supplement brain heart infusion broth at 37°C (input in Fig. 3A) both in unimmunized and RrgB immunized animals. Indeed, the percentage of pilus-1 expressing bacteria in the inoculum was 17% and that observed in the NW ranged from 10.25% to 21%. No difference in pilus expression compared to the inoculum was observed in NW specimens obtained at 24 or 96 hours following challenge. In contrast, reduced pilus-1 expression was observed in the middle ear compared to the inoculum (fig. 3B) in RrgB321 immunized animals suggesting regulation of expression as a mechanism for escape from host defenses.

#### d. Outcome of challenge with Pilus-1 overexpressing mutant in RrgB321 immunized animals

**Figure 6:**
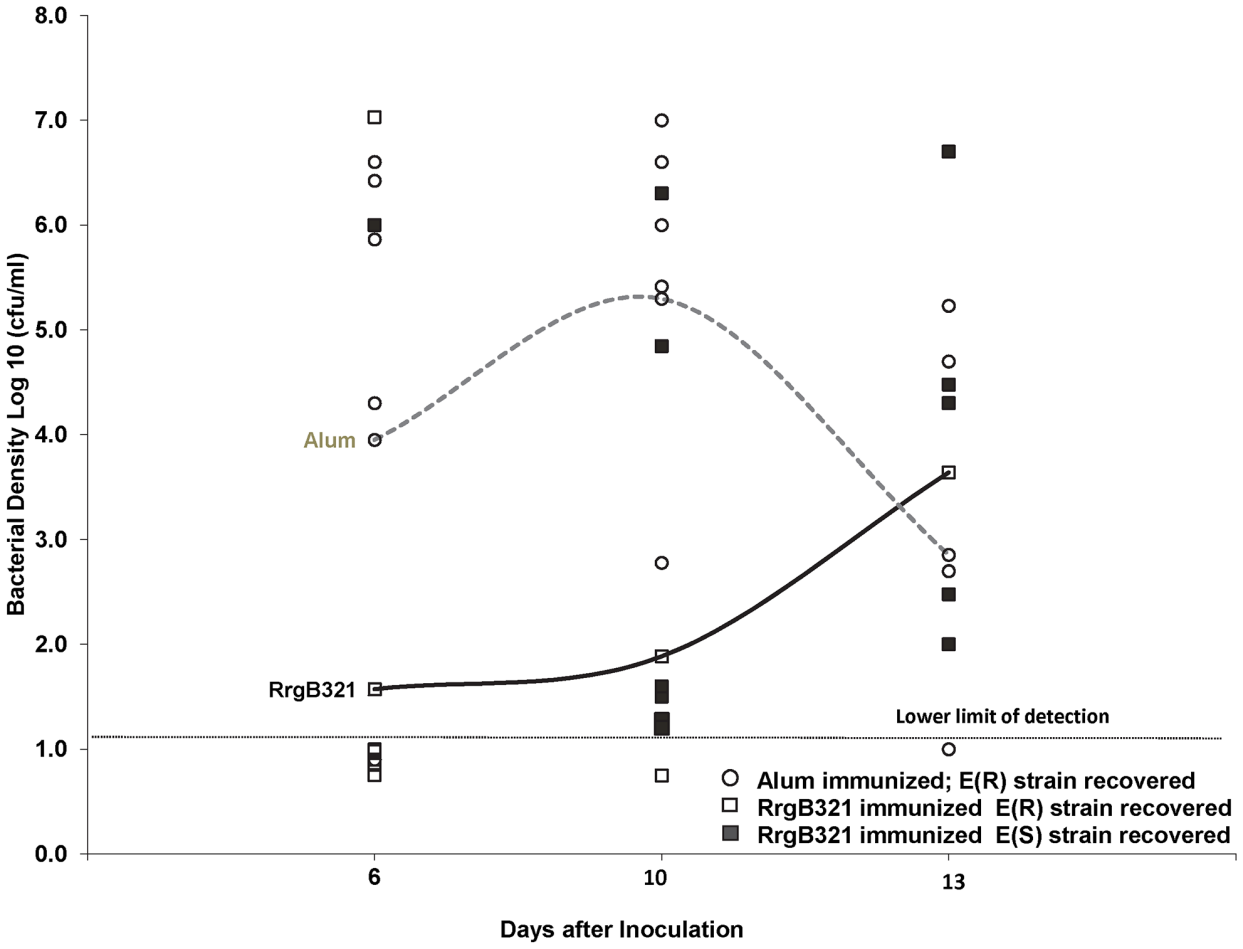
Outcome of challenge with SP Pilus-1 overexpressing mutant in RrgB321 immunized animals. Density of colonization in middle ear fluid in RrgB321 immunized and control chinchillas challenged with SP Taiwan19F-14 pMU1328_*Pc-rlrA* (100% pilus +).

**Figure 7:**
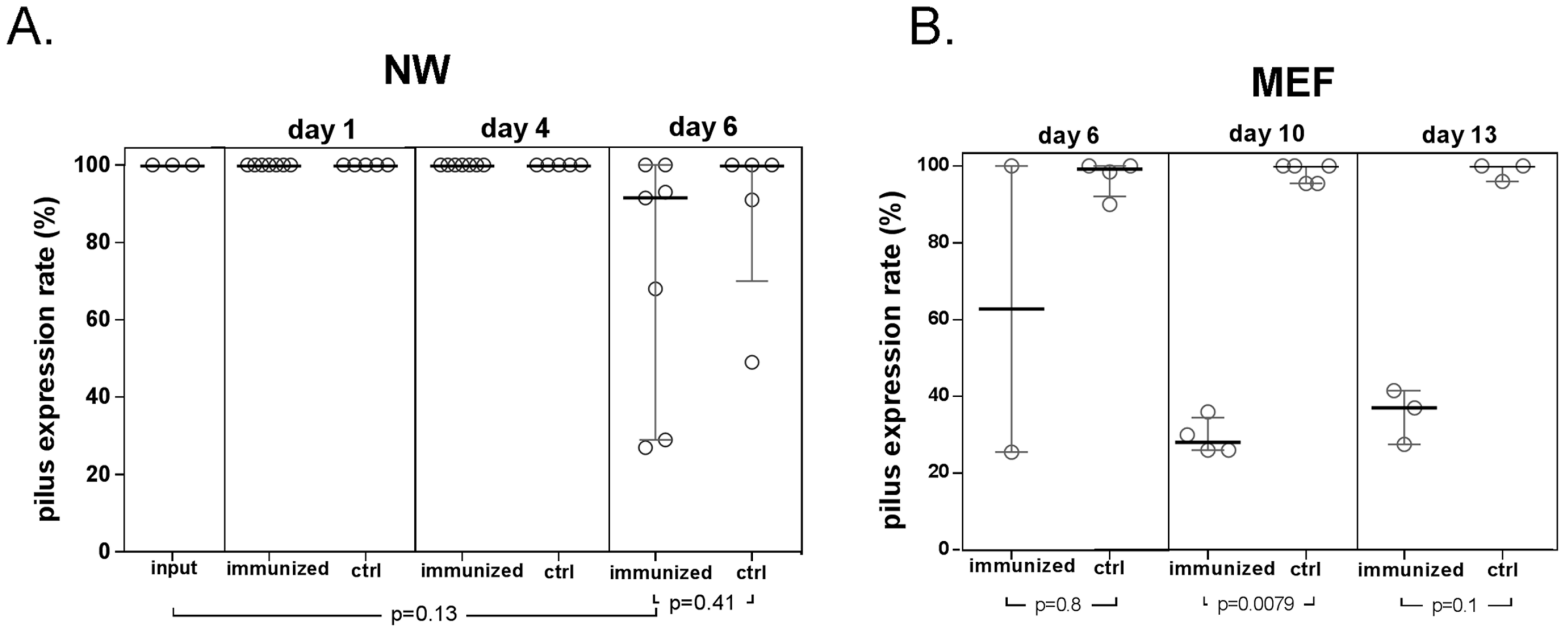
Pilus expression with SP Pilus-1 overexpressing mutant in nasal and middle ear washes of RrgB321 immunized animals. Flow cytometry analysis of Pilus-1 expression in nasal wash (NW) [A] and middle ear Fluid (MEF) [B] in RrgB321 immunized and control animals following challenge with SP Taiwan19F-14 pMU1328_*Pc-rlrA* (100% pilus +).

A second cohort of RrgB321 immunized and control animals were challenged with the over-expressing Pilus-1 mutant. No difference in density of NP colonization was observed between groups and one hundred percent of strains recovered from the NP in each cohort demonstrated pilus expression on days 1 and 4, prior to barotrauma. We observed delayed development of EOM in the immunized animals. All the control animals developed EOM with Pilus-1 over-expressing E(R) mutant SP (open circle) that persisted. In immunized animals, on day 6 after inoculation, only 2 of the 7 animals had developed ME disease; the susceptibility of the SP recovered was different in the two animals, one of the animals had E(R) SP (Open Square) and one E(S) SP (red square). On day 10 after inoculation, 6/7 immunized animals developed ME disease due to E(S) strain and 1 animal did not (Fig. 6). On day 13 after inoculation, five of the 6 surviving animals had culture positive ME disease; the SP recovered from ME fluid was no longer E(R) signifying the loss of the pMU1328_*Pc-rlrA* plasmid. Flow cytometry studies demonstrated reduced pilus expression in those strains that had reverted to erythromycin susceptible confirming the loss of the plasmid (Fig. 7A and B).

## Discussion

We utilized a chinchilla model of experimental otitis media where animals are initially colonized in the nasopharynx by drop inhalation of the inoculum. This strategy prevents aspiration and development of pneumonia and focuses the outcome on otitis media. We compared the outcome of nasopharyngeal challenge with wild type and pilus-1 deficient mutants. As has been reported by others [4] we found the pilus -1 deficient mutant colonized the nasopharynx with non statistically significant reduced densities approximating 1 log at day 4/5, again demonstrating that pilus-1 expression is not essential for colonization. Furthermore, we evaluated the expression of pilus-1 in the nasopharynx in animals colonized with a wild type Taiwan19F-14 strain. Pilus-1 expression was observed in <20% of the bacteria recovered from the nasopharynx on days 1 and 4 despite the fact that a minimum one log expansion of the pneumococcal populations between days 1 and 4 is observed.

One hundred percent of animals challenged with either wild type or the otherwise isogenic pilus-1 deficient mutant developed culture positive EOM within 3 days following barotrauma. The middle ear density in animals challenged with the mutant was ∼1 log less than in those animals challenged with the wild type. Analysis of pilus-1 expression demonstrated that in contrast to the low proportion of pilus expressing strains found in the nasopharynx, up to 80% of the bacteria isolated in animals challenged with the wild type strain expressed pilus-1 on their surface in the middle ear. This observation suggests that the middle ear environment can favor pilus expression despite the ability of non-piliated strains to produce disease. What role pili might play in the pathogenesis of middle ear disease is unclear as infection is primarily extracellular and the relevance of pilus-1 role observed in lung infection may not be comparable to that played in the middle ear compartment. Bacteremia followed initial middle infection in a similar proportion of animals in both disease due to wild type and the pilus deficient mutant. We did not power our animal experiments to determine if subtle differences in time to or density of bacteremia were present.

Our evaluation of the effects due to RrgB321 immunization demonstrated selection against expression of pilus-1 on the bacterial surface in the middle ear of immunized animals. Both immunized and control animals demonstrated low levels of pilus expressing bacteria in the nasopharynx, but in the presence of anti RrgB antibody, upregulation of pilus expression in the middle ear compartment is not observed as occurs in unimmunized animals. Despite the difference in the proportion of pilus expressing bacteria, EOM persists at similar densities in both groups. This observation differs from previous in vitro results obtained by De Angelis and colleagues who found no selection for pilus non expressing bacteria in strains grown in the presence of RrgB hyperimmune rabbit sera. However, our experimental design is considerably different; in fact, the in vitro studies provide a short exposure to diluted antisera and potentially inadequate concentrations of other serum factors that may be necessary for a functional effect. In our model, selection did not appear to occur in the nasopharynx, but was evident in the middle ear first. It is possible that the presence of only low concentrations of antibody and/or complement in the nasopharynx limit functional activity of anti-pilus antibody and selection that becomes evident in the middle ear. These observations may be analogous to the previously described requirement for higher serum anti-capsular antibody concentrations required to prevent serotype specific pneumococcal colonization compared to middle ear or blood stream infection. Following this experimental observation, we hypothesized that if immunized animals were challenged with a *S. pneumoniae* strain genetically engineered to constitutively express pilus-1 in all the bacteria, anti-RrgB antibodies might have been protective. To evaluate this hypothesis, we challenged immunized and control animals with a Taiwan19F-14mutant Erythromycin resistant strain, Taiwan19F-14+pMU1328_*Pc-rlrA*, in which, due to the episomal constitutive expression of the RlrA positive regulator, 100% of the bacteria are positive for pilus-1 expression [2]. Interestingly, high levels of pilus expression (comparable to the input strain) were observed in all the bacteria collected form the nasopharynx of both immunized and control animals at the different time points (all Ery R), thus indicating the maintenance of the plasmid over time in this compartment. Besides, following barotrauma, we found initial protection against EOM in immunized animals challenged with the pilus-1 over-expressing mutant, followed by breakthrough disease with increasing time with bacteria that no longer contained the plasmid as demonstrated by the loss of erythromycin resistance as well as decrease percentage of pilus-1 expressing bacteria in flow studies. Whether this represents selection for a prodigy strain that did not retain the plasmid during replication and then advantaged by the ability to down regulate pilus expression or expulsion of the plasmid in the presence of anti RrgB antibodies cannot be established, even if the former hypothesis seems much more likely.

These observations clearly show, on one side, that vaccine targets which are essential for disease pathogenesis are preferable and, on the other side, the ability of pneumococcus to regulate expression of pilus-1 in the presence of antibody. Croucher reported that rlrA was negatively associated with age in children providing ecologic support to selection against pilus expression as antibodies directed against the pilus components are acquired following repeated pneumococcal colonization [14] Karasic demonstrated that pilus negative strains of nontypable *Haemophilus influenzae* were virulent in an EOM chinchilla model and, similar to our observations, that pilus-expressing strains were selected against when animals were immunized with pilus protein [15]. Clinically, Moschioni et al. reported that 30% of middle ear isolates contained the Pl-1 pilus island, consistent with its distribution among isolates from other sources [16]. Why pilus expression is upregulated in EOM compared to the nasopharynx, yet is not essential for disease, suggests further studies to understand its role in the middle ear are needed as it is likely that pilus expression advantages the pneumococcus in some manner. Recent studies suggest that NTHi can be found intracellulary as part of biofilms in children with persistent and recurrent OM. As suggested for lung disease, perhaps pili have a role in tissue invasion and disease persistence.

These studies suggest that identification of surface proteins as potential candidate vaccine targets must include defining their role in pathogenesis to insure that pneumococci cannot regulate expression to escape vaccine protection. The studies also support a potential role for pilus in the middle ear beyond attachment that appears relevant to its regulation.

## References

[1] Moschioni M, Donati C, Muzzi A, Masignani V, Censini S, et al (2008) Streptococcus pneumoniae contains 3 rlrA pilus variants that are clonally related. J Infect Dis 197: 888–896. 18269316 10.1086/528375

[2] De Angelis G, Moschioni M, Muzzi A, Pezzicoli A, Censini S, et al (2011) The Streptococcus pneumoniae pilus-1 displays a biphasic expression pattern. PLoS One 6: e21269. 21731688 10.1371/journal.pone.0021269 PMC3120856

[3] Pancotto L, De Angelis G, Bizzarri E, Barocchi MA, Giudice GD, et al (2013) Expression of the Streptococcus pneumoniae pilus-1 undergoes on and off switching during colonization in mice. Sci Rep 3: 2040. 23784148 10.1038/srep02040 PMC3687230

[4] Rosch JW, Mann B, Thornton J, Sublett J, Tuomanen E (2008) Convergence of regulatory networks on the pilus locus of Streptococcus pneumoniae. Infect Immun 76: 3187–3196. 18443093 10.1128/IAI.00054-08 PMC2446684

[5] Bagnoli F, Moschioni M, Donati C, Dimitrovska V, Ferlenghi I, et al (2008) A second pilus type in Streptococcus pneumoniae is prevalent in emerging serotypes and mediates adhesion to host cells. J Bacteriol 190: 5480–5492. 18515415 10.1128/JB.00384-08 PMC2493256

[6] Bouchet V, Hood DW, Li J, Brisson JR, Randle GA, et al (2003) Host-derived sialic acid is incorporated into Haemophilus influenzae lipopolysaccharide and is a major virulence factor in experimental otitis media. Proc Natl Acad Sci U S A 100: 8898–8903. 12855765 10.1073/pnas.1432026100 PMC166410

[7] Alloing G, Martin B, Granadel C, Claverys JP (1998) Development of competence in Streptococcus pneumonaie: pheromone autoinduction and control of quorum sensing by the oligopeptide permease. Mol Microbiol 29: 75–83. 9701804 10.1046/j.1365-2958.1998.00904.x

[8] Achen MG, Davidson BE, Hillier AJ (1986) Construction of plasmid vectors for the detection of streptococcal promoters. Gene 45: 45–49. 3536665 10.1016/0378-1119(86)90130-7

[9] Sabharwal V, Ram S, Figueira M, Park IH, Pelton SI (2009) Role of complement in host defense against pneumococcal otitis media. Infect Immun 77: 1121–1127. 19139190 10.1128/IAI.01148-08 PMC2643640

[10] Moschioni M, De Angelis G, Harfouche C, Bizzarri E, Filippini S, et al (2012) Immunization with the RrgB321 fusion protein protects mice against both high and low pilus-expressing Streptococcus pneumoniae populations. Vaccine 30: 1349–1356. 22210141 10.1016/j.vaccine.2011.12.080

[11] Sabharwal V, Figueira M, Pelton SI, Pettigrew MM (2012) Virulence of Streptococcus pneumoniae serotype 6C in experimental otitis media. Microbes Infect 14: 712–718. 22414497 10.1016/j.micinf.2012.02.008 PMC3382049

[12] Babl FE, Pelton SI, Li Z (2002) Experimental acute otitis media due to nontypeable Haemophilus influenzae: comparison of high and low azithromycin doses with placebo. Antimicrob Agents Chemother 46: 2194–2199. 12069974 10.1128/AAC.46.7.2194-2199.2002 PMC127299

[13] Figueira MA, Ram S, Goldstein R, Hood DW, Moxon ER, et al (2007) Role of complement in defense of the middle ear revealed by restoring the virulence of nontypeable Haemophilus influenzae siaB mutants. Infect Immun 75: 325–333. 17088344 10.1128/IAI.01054-06 PMC1828410

[14] Croucher NJ, Finkelstein JA, Pelton SI, Mitchell PK, Lee GM, et al (2013) Population genomics of post-vaccine changes in pneumococcal epidemiology. Nat Genet 45: 656–663. 23644493 10.1038/ng.2625 PMC3725542

[15] Karasic RB, Beste DJ, To SC, Doyle WJ, Wood SW, et al (1989) Evaluation of pilus vaccines for prevention of experimental otitis media caused by nontypable Haemophilus influenzae. Pediatr Infect Dis J 8: S62–65. 2564659 10.1097/00006454-198901001-00021

[16] Moschioni M, De Angelis G, Melchiorre S, Masignani V, Leibovitz E, et al (2010) Prevalence of pilus-encoding islets among acute otitis media Streptococcus pneumoniae isolates from Israel. Clin Microbiol Infect 16: 1501–1504. 19886901 10.1111/j.1469-0691.2009.03105.x

[17] Shi ZY, Enright MC, Wilkinson P, Griffiths D, Spratt BG (1998) Identification of three major clones of multiply antibiotic-resistant Streptococcus pneumoniae in Taiwanese hospitals by multilocus sequence typing. J Clin Microbiol 36: 3514–3519. 9817864 10.1128/jcm.36.12.3514-3519.1998 PMC105231

